# Traits and types of health data repositories

**DOI:** 10.1186/2047-2501-2-4

**Published:** 2014-06-30

**Authors:** Ted D Wade

**Affiliations:** Division of Biostatistics and Bioinformatics, National Jewish Health, Denver, CO 80206-2761 USA

**Keywords:** Registry, Observational research, Big data, Information commons, Data warehouse, Federated database

## Abstract

We review traits of reusable clinical data and offer a typology of clinical repositories with a range of known examples. Sources of clinical data suitable for research can be classified into types reflecting the data’s institutional origin, original purpose, level of integration and governance. Primary data nearly always come from research studies and electronic medical records. Registries collect data on focused populations primarily to track outcomes, often using observational research methods. Warehouses are institutional information utilities repackaging clinical care data. Collections organize data from more organizations than a data warehouse, and more original data sources than a registry. Therefore even if they are heavily curated, their level of internal integration, and thus ease of use, can be less than other types. Federations are like collections except that physical control over data is distributed among donor organizations. Federations sometimes federate, giving a second level of organization. While the size, in number of patients, varies widely within each type of data source, populations over 10 K are relatively numerous, and much larger populations can be seen in warehouses and federations. One imagined ideal structure for research progress has been called an “Information Commons”. It would have longitudinal, multi-leveled (environmental through molecular) data on a large population of identified, consenting individuals. These are qualities whose achievement would require long term commitment on the part of many data donors, including a willingness to make their data public.

## Introduction

Recent years have seen repeated calls [1–4] to make better use of both existing and future biomedical data in order to more quickly and economically advance research and patient care. This is accompanied by advocacy for non-experimental designs and techniques that improve the validity of using existing observations in research [5, 6], and for innovative exploratory or data mining [7–9] studies. There is heightened awareness of problems [10] such as cultural or legal barriers to data sharing, and of technical problems of reuse, such as lack of syntactic or semantic comparability of data, or poor accessibility or preservation of data.

Such critiques can give the impression that suitable clinical, phenotypic data of appropriate volume and condition are difficult to locate. This paper counters that hypothesis by offering a sampling of major clinical/phenotypic sources, along with a classification system to help would-be users understand more about general issues of availability and usability. The review is mostly focused on sources in the United States, where complex delivery and research systems are accompanied by a variety of data repository solutions.

## Repository traits and types

**Figure 1:**
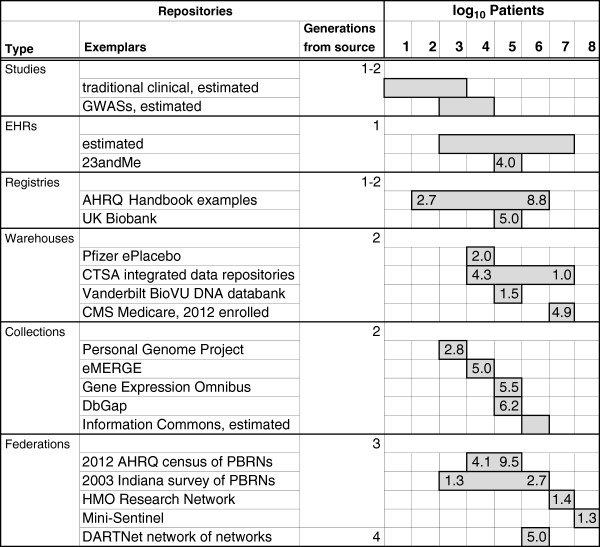
**Biomedical repository types and sizes.** Each type has exemplars with size or range of sizes shown as the log_10_ of the number of distinct patients represented. When a cell has a number, it is the coefficient of the log: e.g., a 2.7 in the 2 column means 2.7 × 10^2^. A filled cell with no number is either part of a known range, or part of an order of magnitude estimated range. *Generations from source* refers to generations of modification of data or access methods, where the original source data is generation 1. Types and exemplars are discussed in the text. Specific exemplars only appear if data for estimating their size are available.

Descriptions of the sources will refer to repository traits (Table 1) that make them more or less useful and available for research. The first two traits are quantitative ones that we use later (Figure 1) to compare all the repository types. The first trait is the number of patients or research subjects observed. Users of our own data warehouse have made it clear that the number of potential patients is a primary concern for researchers when judging whether a data source is useful. Though in Figure 1 these numbers vary across eight orders of magnitude, they still fail to capture much of the variability of data volume because the numbers of observations made on each patient also vary widely. For strictly clinical databases, the number of observations per patient might vary from the 10’s to the low 1000’s. For biomolecular data, typical data points per patient are often in the 10 K’s to 100 K’s. The range is much wider: from a low end of highly focused assays to a high end of sequencing entire genomes. Even being conservative, the number of data objects one might find in a re-usable clinical database right now could vary from roughly 10^2^ for a clinical pilot study to 10^11^ (our estimate for DbGap [11]) -- a staggering range.

**Table 1:** **Data repository traits that are relevant to data reuse**

Repository trait	Definition
Sample size	Number of patients represented.
Data generations from source	Number of times data or access methods were modified, where generation 1 is original source data.
Level of integration	Extent of structuring that organizes data for query.
Longitudinal observations	Containing observations over multiple times per patient.
Personally identified	Capable of delivering direct patient identifiers to research projects.
Research accessibility	Extent to which data are accessible to researchers, whether within or outside of a home institution.
Data quality	Accuracy, completeness and consistency of data expression.
Linked biosamples	Having available biosamples linked to phenotypic information.
Biomolecular data	Having biomolecular/omics data linked to phenotypic information.

Because reuse of data nearly always requires some re-organization and curation, our second dimension of the resources (Table 1) is the number of generations that data are removed from their primary sources. The first generation is “primary” data -- as first recorded for clinical or research purposes. Subsequent generations modify data or its access methods successively, up to a fourth generation for networks of federated data networks.

Data repositories vary in their levels of integration (Table 1), i.e., the extent of internal organizing structures that provide such functions as: locations, indices, catalogs, semantic translations or equivalences, consistent syntactic structures and links to external information [12]. The level of integration of a data repository affects its usefulness by constraining how readily one may make a detailed global query for any subset of its contents. Integration structures that support one use case for a repository may not support other use cases as well. It is often hard to learn about the level of integration of a data source until you start to use it.

Having longitudinal data on individuals is a part of many observational designs, and is needed for research into outcomes, efficacy and many mechanistic studies, [2, 3]. Most repositories thus have longitudinal observations (Table 1). To build such a database you need some way to link observations on the same identified person. Therefore most repositories contain personally identified data, but, because of privacy concerns, they often release only de-identified data. Difficulties in the de-identification process can cause some data to be omitted in a dataset [10]. A lack of direct identifiers in a data collection or federation (terms defined in Table 2) could prevent linking of data for some patients [13].

**Table 2:** **Types of clinical data repositories**

Repository type	Definition
Study	A database that collects observations for a specific clinical research study.
EHR	A database of observations made as a result of direct health care.
Registry	Observations collected and organized for the purpose of studying or guiding particular outcomes on a defined population. Associated studies are either multiple or longterm and evolving over time.
Warehouse	A repository that adds levels of integration and quality to the primary (research or clinical) data of a single institution, to support flexible queries for multiple uses. Is broader in application than a registry.
Collection	A library of heterogeneous data sets from more organizations than a warehouse or more sources than a registry. Organized to help users find a particular data set, but not to query for data combined across data sets.
Federation	A repository distributed across multiple locations, where each location retains control over access to its own data, and is responsible for making the data comparable with the data of other locations.

Repositories vary based on the purpose, origin, control and integration of their data.

Data repositories vary in how they restrict which researchers, or which types of research, can use them (Table 1). While some are fairly open, others are restricted either to employees of an institution or to members of some research network. Thus a researcher’s access to data riches might require a collaboration with someone who has direct access.

Data quality (Table 1) and its fitness for a particular purpose is a multidimensional problem [14] and a common concern for data reuse [13]. Users of a repository need to be aware of the data’s original purpose and of what has been done to improve its fitness for reuse or being compared with other data.

Some repositories gain value by having biosamples available and linked to clinical data [15]. Others gain value by linking biologically derived data to clinical observations [11, 16] (Table 1).

The US (United States) National Institutes of Health recently announced a “Big Data to Knowledge” (BD2K) initiative [17]. How big are available resources, and what makes them useful? We try to address this by a typology of repositories (Figure 1) based on how and why they are constructed (Table 2). The classifications are not absolute because one could readily classify some data repositories in more than one category.

## Studies and EHRs

The first generation databases in Figure 1 are of two types: databases collected for individual research studies, and electronic health records (EHRs). As we estimate in Figure 1 the size range of EHRs is wide, since it includes installations in small medical practices and long-term databases in large healthcare organizations. The structure and function of EHRs tends to limit their direct use in research, so EHR data used in research is typically extracted first into a registry or data warehouse (see below). Repositories of personal electronic health records exist, and we include an example, the genome-centered 23andMe, in which 90% of subjects allow some research use of their data [18].

In Figure 1 we estimate clinical studies to have sizes from the 10’s to 1000’s of patients. Research study data have generally higher quality, in terms of consistency and completeness, due to the elaborate processes used to assure this [2, 3]. However, their data semantics (the meaning of the observations) may often be narrow and poorly suited for comparison to other data [19]. Research data are very expensive to acquire, practically limiting the sample size, but genome-wide association studies have tended to raise sample sizes. Research studies can sometimes include some second-generation data derived from regular clinical care.

## Registries

In its comprehensive handbook on building *registries* the Agency for Healthcare Research and Quality (AHRQ) says ([5] page 1) that “A *patient registry* is an organized system that uses observational study methods to collect uniform data (clinical and other) to evaluate specified outcomes for a population defined by a particular disease, condition, or exposure, and that serves one or more predetermined scientific, clinical, or policy purposes”. Registries thus exemplify observational methods in their construction and use. They are often of hybrid generation (both first and second) because they might include primary data from other sources. The purposes served by a registry can be quite broad, and -- because their data are of higher quality than routine clinical care observations -- they could potentially be used for purposes that were not part of their original plan. Vandenbrouke [6] noted that collecting data for prospective follow-up studies (a typical registry purpose) “is so huge an undertaking for the study of causes of disease that researchers only begin such investigations when they are really necessary to confirm or refute something important”. Thus the reuse of expensive, high-quality registry data seems like a good idea.

The size of registries varies a lot. The size of the 36 registries used as examples in the comprehensive AHRQ Handbook [5] ranged over 5 orders of magnitude, with 61% of them in the range from 1 K to 100 K patients. The UK Biobank [20] is essentially a massive, very general-purpose registry of 500 K patients and their biospecimens that has recently become available for research. It is expensive enough to require support from multiple governments, a major charity and a major foundation.

## Warehouses

Clinical data *warehouses* are repositories of information from clinical, and sometimes research, records from a single organization, such as a care provider or a payer. A warehouse normally has a high level of integration to allow very flexible queries of its content. They typically have the ability to de-identify queried data, or to allow query for frequencies of records. Both are methods making researcher access easier [21]. Warehouses operate [22] as sort of an information utility for their host institutions, allowing cohort discovery for prospective research (both observational and experimental) and supporting retrospective queries for quality of care work, pilot studies, and case–control research. As a second-generation data source, warehouses attempt to standardize their incoming data for more effective use [22], in a process known generically in the database industry as ETL, meaning “extract, transform, and load”. The growth of clinical data warehouses has benefitted greatly from having a standard, publically-available platform called i2b2 [23]. The i2b2 platform has crossed over to also be used in building registries [24].

A 2010 survey [21] of academic health centers receiving U.S. Clinical and Translational Science Awards found 22 institutional data warehouses in 35 institutions. The number of patients in a warehouse (Figure 1) ranged from 43 K to 10 M, with a median of 1.6 M. These numbers reflect the generally large clinical patient populations at many research hospitals.

Vanderbilt’s BioVU DNA data warehouse is noteworthy because of its incorporation of a biobank and derived genomic data [25]. BioVU links more than 150,000 unique genetic samples [26] to de-identified medical records. Part of its successful growth is due to having patient participation be decided on an opt-out basis, a new approach that is said to satisfy ethical concerns while increasing diversity of the research population, and thus wider application of research’s benefits [25]. The resource has been used for over 15 genome-wide association studies, and for correlation of adverse drug effects with genome scans.

Even larger, in fact the largest (in terms of patients) *single* database of any kind in this review is the US Centers for Medicare and Medicaid Services (CMS) database [27] of inpatient and outpatient data on citizens over 65. Figures for enrollment varied from 41 M in 2002 to 49 M in 2012 [28]. This is payer, not clinical, data but includes much useful detail on diagnoses, demographics, the services provided and the costs of those services. The comprehensive nature of the data, especially for hospitalization, makes this database very useful for longitudinal outcomes research. The database is being restructured with “big data” technology [29] to increase its usability and capacity.

## Collections

A *collection* is a data repository that combines, into one location, data that originated from multiple independent sources. So a collection involves more organizations than a data warehouse and/or more original data sources than a registry. The term, collection, was chosen by analogy with holdings in a library, with a catalog (the metadata) holding the collection together and focusing access on the individual books or journals. The usual heterogeneity of datasets in a collection means that their structure, codings and meanings may make them less integrated or comparable than data in warehouses or registries. It may be quite impractical to index collection data so that, for example, a researcher could search 1000 different studies for the values of a particular type of laboratory test. When such integration is not practical it is instead useful to standardize and index *metadata* – e.g., whether a study is known to be a randomized controlled trial, or whether it has single nucleotide polymorphism data. The metadata catalog at least lets a database user know that by examining selected studies’s data separately they can continue to search for needed details.

Prototypical collections (Figure 1) are the Gene Expression Omnibus (GEO) [30] and the NIH’s Database of Genotypes and Phenotypes, dbGaP [11]. GEO is a large international collection of functional genomics data, including data from many species and studies with many purposes. The data on the approximately half a million humans are de-identified, with considerable variability in their phenotypic or experimental characteristics, but still very much usable. This usability was shown, for example, in a large disease/gene expressions correlation study [31]. dbGaP is human and genotype-focused, with more emphasis on useful, searchable detail about phenotypes. But it is also a collection because search is still a hierarchical, multi-step, affair.

The eMERGE (Electronic Medical Records and Genomics) Network [15] collects biosamples, SNP data, and phenotypes computed from EMR data, all from a national consortium of 10 institutions. The eMERGE collection is more highly curated, with an integration level more like a data warehouse. The integration includes the development of algorithms to extract standard phenotypes (e.g., [32]) from disparate medical record systems. The facility supports a number of different collaborations, so its data structures are optimized for those. But it also allows members to query across the entire subject population and obtain counts of subjects having combinations of ICD9 and CPT codes, stratified by demographics (personal communication). Note that compared to the collections mentioned above, eMERGE is an order of magnitude smaller, possibly reflecting the cost of its higher level of integration.

## Federations

Data federations are somewhat like collections except that a larger share of control remains with the organizations contributing their data. Usually data do not leave those organizations, except in summary form, when someone queries the federation for data. The informatics work to make data comparable across the network is itself distributed among the participant organizations. Thus each participant needs to re-organize their primary data for sharing. After this, the addition of a step to aggregate information across the network means that federations normally use third-generation data. This 3-step process (EHR to warehouse to federation) was also described as prototypical by Kahn and Weng (13). The gigantic (and paradoxically named) Mini-Sentinel federation for product safety surveillance [33] is our largest example of any data system, but its possible use outside of its designed purpose is still undetermined.

Another very large federation is the HMO Research Network (HMORN) [34] of 18 health maintenance organizations, covering 14 M patients in the United States and Israel. A sub-network of the HMORN is the Kaiser Permanente consortium, which is reported to have over 9 M patients [35]. The HMORN shares [36] with Mini-Sentinel a technology base, PopMedNet [37] that allows data holders to independently decide whether to respond to particular queries from the network. HMORN exemplifies how research networks grow and intersect. Members of HMORN have collaborated on hundreds of peer-reviewed projects, with varying partners in and out of the network [38]. Currently HMORN members participate in at least 10 consortia, each involving at least 10 or more members, some including non-network partners as well. In fact there are 13 HMORN members out of the 33 organizations in Mini-Sentinel.

One type of data federation is rather common, taking the form of “practice-based research networks” (PBRNs) of small to medium ambulatory care providers that are [39] “laboratories for primary care research and dissemination” (see, e.g. [40]). In Figure 1 we show two inventories of PBRNs. A 2003 survey [41] by Indiana University contacted 86 PBRNs representing 1,871 practices covering a total of 14.7 M patients. Most were open to research initiated by non-members. A 2012 [39] inventory by the US Agency for Healthcare Research and Quality (AHRQ), which strongly encourages PBRNs and supports them in various ways, counted more than 150 networks, at over 17 K locations, with 55 K clinicians serving about 46 M patients. Those counts were used in Figure 1 to make a rough estimate of the range of patient numbers per PBRN. There seems to be growth in the number and coverage of PBRN networks in the US over a decade, and the number of available patients is very large.

The evolution of PBRNs includes networks of networks, which means fourth generation data. The one example shown (Figure 1) out of several found, DARTNet [42], is a federation that contains 9 distinct networks covering 5 million patients. As with the growth of the HMORN meta-network, evolution into larger or second-order federations is facilitated by adoption of common technology or data models.

## The information commons as a super-collection

The collection concept has been taken to something like an ultimate conclusion with the recent US Institute of Medicine proposal [3] for a national *Information Commons and Knowledge Network*. This proposes to build a giant database from data collected during normal medical care. It would bypass the clinical trials bottleneck to produce breakthrough efficiency in building the knowledge needed for individualized, precision medicine. Among its critical features would be:

- Massive size and high levels of integration.
- An “individual-centric” Information Commons - linking data from *multiple phenotypic and environmental levels* (clinical, exposures, genome, epigenome, microbiome, etc.) by retention of *longitudinal* data on identified individual patients.
- Gradual growth to solve structural, security, and regulatory/governance issues while incorporating new types of data as they become part of regular clinical care.
- Feedback of knowledge obtained from derived studies and the general literature into a Knowledge Network.

The committee said that only a “massive re-orientation of … information systems” *to vertical integration of data on individuals* will allow the kinds of research needed. In Figure 1 we assume that the Information Commons would be at most an order of magnitude larger than existing large collections like dbGaP or GEO because of the system integration and governance costs. The report’s strong position for identified data linkage contrasts with a workshop [10] from *the same* Institute of Medicine on research data sharing. The latter continues with the common assumption that de-identification of data is necessary before collection and reuse. However they did note that patient engagement in, awareness of, and sharing the benefits of, research needs to increase for multiple reasons, not the least being respect for patient dignity.

How might the “massive re-orientation” that is needed for the Information Commons evolve? The authors [3] noted that the development of dbGap – even though it is de-identified and less integrated -- is evidence that the Information Commons project is possible. One research organization, Sage Bionetworks [43], has developed a “Portable Legal Consent” to enable patients, with one document, to donate any of their data at any time. The consent is being used with their open, web-based platform called BRIDGE, creating small disease-focused communities of patients to prototype “citizen participation” in multi-leveled research. The organization is also supplying a free data federation platform, Synapse, for researcher collaboration. Synapse has already enabled an instance of unprecedented cooperation and data sharing [44] among 60 different research projects in The Cancer Genome Atlas program.

The Information Commons would need continuing and comprehensive coverage on data from a wide variety of patients. Because data in a person’s clinical history accumulate over time and from different sources, new information can only be linked by matching on individual identifiers. Research studies would not necessarily need access to those identifiers as long as all data on each individual were linked. However, the inclusion of detailed genetic information would mean that re-identification of research data would be feasible [45]. Therefore a data donor would have a significant risk of loss of confidentiality.

We already know that patients will share personal health information in certain circumstances, such as when they have debilitating chronic diseases (e.g., PatientsLikeMe [46]). Quite a few people will also entrust private health information to a third party repository [18] and even allow some research on their data. So the question for the Information Commons is: would enough people donate nearly all of their identified health data on a continuing (longitudinal) basis to be used in future research of any legitimate type? To obtain the needed patient consents and participation there would have to be an educational effort to promote the values of personal data sharing -- for which appealing arguments [43, 45] have been advanced. So far the only repository of public, identifiable data, both genomic and phenotypic, is the Personal Genome Project, which is also a biorepository. The number of participants is still fairly low at 2,781 (Figure 1 and [16]).

Whether we are at “a tipping point and historic moment of scientific opportunity” [43] to build the Information Commons may depend on how readily the concept spreads and acquires resources that might have otherwise gone towards more traditional approaches to research. One sign in the Commons’s favor is the recent US Patient-Centered Outcome Research Institute’s plan [47] to construct a large federation called the “PCORnet National Patient-Centered Clinical Research Network”, to be used for both observational and interventional comparative effectiveness studies and possibly for other types of research, all at a national scale. Data will be longitudinal and ongoing. One component of the network is a set of existing large health systems and federations, called CDRNs, who will contribute large amounts of routine clinical care data, suitably curated to a standard. The other component is a set of diverse patient-centered networks called PPRNs that are focused on particular disease conditions, some of them rare. These will collect both clinical and patient-generated data, with a focus on patient involvement (“patient-centered”). In our terminology, the PPRNs will be like a federation of registries, but also having a connection to the CDRN federation of federations. PCORnet is tackling big issues relevant to the formation of an Information Commons, including governance, privacy, standardization, sustainability and patient involvement in, and consent for, research. For PCORnet to be a nucleus for growth of the Commons would also require the eventual addition of biological levels of data, as well as wider consent to make personal data public.

## Conclusions

Research and clinical care have provided large and growing pools of reusable clinical data in a variety of institutional and public settings. The majority have subject populations of at least 10 K, with quite a few having 1–4 orders of magnitude more. The utility of these repositories varies: in the levels of internal organization that affect their usability, in whether they also contain linked tissue or bio-molecular data, and in the extent to which they are accessible to non-affiliated researchers. The ideal data source, described by some as an Information Commons, could only be achieved by advances in regulation, technology and public opinion. Its proponents think that it would enable a breakthrough in the progress of biomedical research and its application to human health.
